# Adropin Is Expressed in Pancreatic Islet Cells and Reduces Glucagon Release in Diabetes Mellitus

**DOI:** 10.3390/ijms25189824

**Published:** 2024-09-11

**Authors:** Ifrah I. Ali, Crystal D’Souza, Saeed Tariq, Ernest A. Adeghate

**Affiliations:** 1 Department of Anatomy, College of Medicine & Health Sciences, United Arab Emirates University, Al Ain P.O. Box 15551, United Arab Emirates; 200540058@uaeu.ac.ae (I.I.A.); crystal.dz@uaeu.ac.ae (C.D.); stariq@uaeu.ac.ae (S.T.); 2 Zayed Foundation, United Arab Emirates University, Al Ain P.O. Box 15551, United Arab Emirates

**Keywords:** diabetes mellitus, rats, adropin, pancreatic islets, insulin, glucagon, immunohistochemistry, electron microscopy, Western blot, receptors

## Abstract

Diabetes mellitus affects 537 million adults around the world. Adropin is expressed in different cell types. Our aim was to investigate the cellular localization in the endocrine pancreas and its effect on modulating pancreatic endocrine hormone release in streptozotocin (STZ)-induced diabetic rats. Adropin expression in the pancreas was investigated in normal and diabetic rats using immunohistochemistry and immunoelectron microscopy. Serum levels of insulin, glucagon pancreatic polypeptide (PP), and somatostatin were measured using a Luminex^®^ χMAP (Magpix^®^) analyzer. Pancreatic endocrine hormone levels in INS-1 832/3 rat insulinoma cells, as well as pancreatic tissue fragments of normal and diabetic rats treated with different concentrations of adropin (10^−6^, 10^−9^, and 10^−12^ M), were measured using ELISA. Adropin was colocalized with cells producing either insulin, glucagon, or PP. Adropin treatment reduced the number of glucagon-secreting alpha cells and suppressed glucagon release from the pancreas. The serum levels of GLP-1 and amylin were significantly increased after treatment with adropin. Our study indicates a potential role of adropin in modulating glucagon secretion in animal models of diabetes mellitus.

## 1. Introduction

Diabetes mellitus (DM) is a major health issue that has reached alarming levels. It is one of the largest global public health concerns as it is considered a leading cause of mortality and has a major impact on individuals’ well-being worldwide. According to the International Diabetes Federation, the global diabetes prevalence in 2021 was estimated to be 537 million people around the world, which makes up 10.5% of the global adult population between the ages of 20 and 79 years. By 2030, it is speculated that this number will increase to 643 million, and 783 million by 2045 [1]. The distressing fact is that there was a 125.5% increase in global deaths due to diabetes from 1990 to 2017.

DM is defined as a chronic disease, characterized by hyperglycemia with disturbances of carbohydrate, fat, and protein metabolism resulting from either a defect in insulin secretion by the pancreas or impairment in insulin action in insulin-sensitive tissues, or both [2]. There are two main types of diabetes mellitus: type 1 diabetes mellitus (T1DM) and type 2 diabetes mellitus (T2DM). In T1DM, the body attacks its own pancreatic β-cells, leading to defects in these cells and depletion in insulin production [3]. On the other hand, T2DM is described as a low response to insulin by insulin-sensitive tissues, and it is characterized by insulin action disorder and β-cell dysfunction. This type of diabetes is frequently undiagnosed for many years because the hyperglycemia is often not severe enough to induce noticeable symptoms at the first onset [2]. Other than β-cell dysfunction, pancreatic α-cells are also disrupted in diabetes mellitus. Due to the reduction in β-cell mass in diabetes, the paracrine regulation by β-cells on glucagon secretion from pancreatic α-cells is defective, leading to hyperglucagonemia [4]. Impairment in insulin action and glucose sensing in the liver and muscle may also cause dysfunction in glucagon release [5]. Additionally, expansion of α-cell mass, increased cell proliferation, and elevated plasma glucagon levels were reported in diabetes mellitus [6,7]. The biggest challenge in diabetes mellitus is maintaining blood glucose levels within the normal range and avoiding diabetic complications. The key is to follow proper management of diabetes, whether it is lifestyle modifications or therapeutic management. Several peptides, such as insulin and incretin mimetics, have been used as therapeutic options for diabetes mellitus [8,9]. A recently discovered peptide, known as adropin, has shown a promising result in fighting diabetes. Adropin is a short peptide consisting of 76 amino acids, encoded by the *Enho* gene [10]. It has been reported that this peptide acts on the orphan G-protein-coupled receptor 19 (GPR19) [11]. Adropin’s early discovery proved that the peptide is expressed in the liver [10] and brain [12]. The tissue distribution of adropin has since been demonstrated in a variety of tissues and cells. For instance, adropin was detected in pancreatic acinar cells as well as capillaries of pancreatic islets [13,14]. Moreover, adropin was found in the endothelial cells of the circulatory system, renal glomeruli, and in the epicardium, myocardium, and endocardium of rat hearts [13,15,16].

Adropin plays a role in regulating lipid and glucose metabolism, and its circulating level is modulated by nutrient consumption [17]. It is worth mentioning that adropin levels can be affected by metabolic disorders, such as obesity and diabetes. A lower serum adropin concentration was detected in patients with T2DM and in children with T1DM, in comparison to healthy controls [18,19]. In addition to that, the risk of developing diabetic complications has been positively correlated with low levels of adropin [20]. In contrast, human and animal studies have reported higher adropin levels in diabetic subjects [13,21]. Kumar et al. demonstrated a direct association between adropin deficiency and insulin resistance and increased adiposity [22]. This opened a new window to investigating the potential role of adropin in diabetes mellitus management. Thereafter, several studies have been conducted in this field to test its association with diabetes mellitus. Thapa et al. reported reduced fasting blood glucose after the administration of exogenous adropin to mice fed with a high-fat diet, suggesting the capability of adropin in suppressing hepatic glucose production [23]. Moreover, there is overwhelming evidence that adropin may contribute to increased hepatic insulin sensitivity, enhance cardiac function and cardiac energy metabolism, and regulate glucose utilization in skeletal muscles [24,25,26]. However, little is known about the role of adropin in the pancreas of diabetic subjects and whether it can modulate insulin and glucagon release.

**Figure 1:**
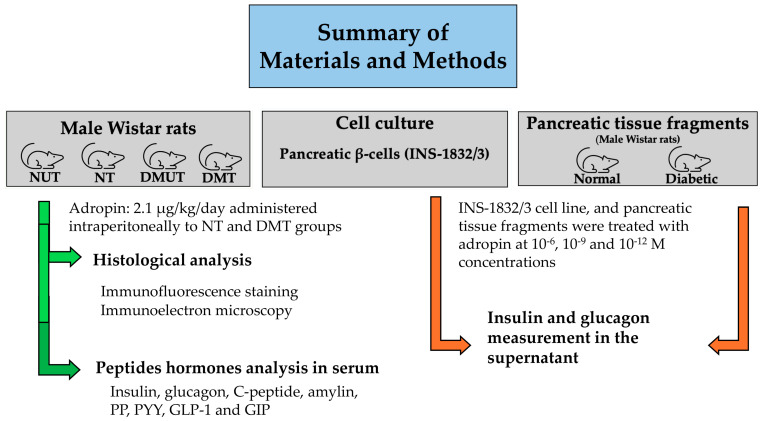
A flowchart illustrating the materials and methods for this study. NUT = normal untreated; NT = normal treated; DMUT = diabetic untreated; DMT = diabetic treated. PP = pancreatic polypeptide; PYY = peptide YY; GLP-1 = glucagonlike peptide 1; GIP = gastric inhibitory peptide.

Based on the previous findings, we hypothesized that adropin treatment would improve metabolic parameters associated with diabetes and enhance pancreatic endocrine hormone secretion in the pancreas of diabetic rats. Our aims in this study were to investigate the pattern of distribution of adropin in the endocrine pancreas of normal and diabetic rats and to determine whether adropin can modulate insulin and glucagon release. Our specific objectives were to investigate the effect of adropin on body weight and fasting blood glucose and to study adropin expression and tissue distribution in pancreatic islet cells (α, β, d, and PP cells) in normal and diabetic rats. Another goal was to investigate how adropin could affect the secretion of the endocrine pancreatic hormones as well as other hormones involved in glucose metabolism. A streptozotocin-induced diabetic model, similar to that of T1DM, was used because we wanted to examine how adropin would affect the plasma levels of insulin and glucagon, the major hormones that are altered after the onset of DM. Appropriate methodology was followed to achieve our objectives (Figure 1).

## 2. Results

### 2.1. Adropin Level and Expression of GPR19 Receptor in the Pancreas of Normal and Diabetic Rats

**Figure 2:**
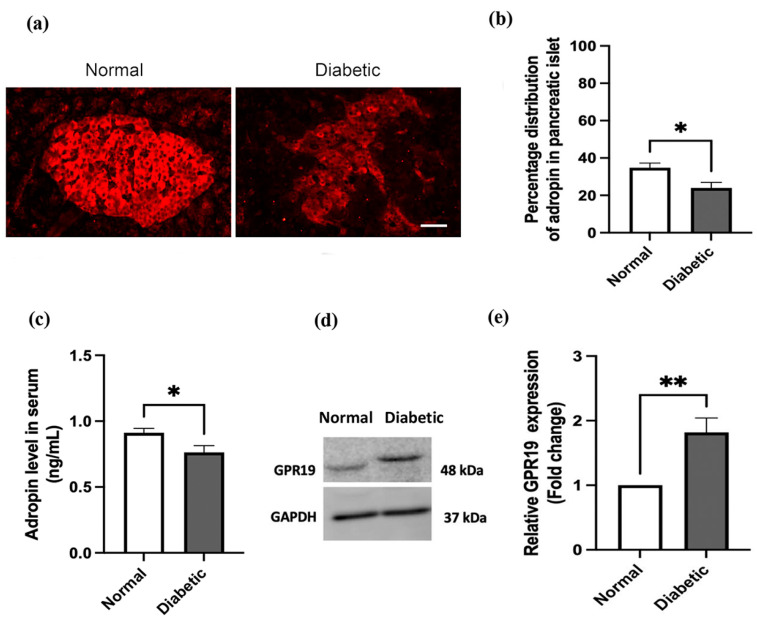
Adropin peptide and GPR19 expression in the pancreas of normal and diabetic rats. Immunofluorescence staining using anti-adropin antibody and secondary antibody conjugated with TRITC (red). (**a**). Adropin protein expression was significantly lower in diabetic compared to normal rats. (**b**). Morphometric analysis of the immunostaining of adropin in (**a**) showed that the number of adropin-positive cells was significantly reduced after the onset of diabetes. (**c**). The serum level of adropin is significantly reduced in the diabetic group. (**d**). GPR19 was significantly higher in diabetic compared to normal rats. Uncropped images of the blots for Figure 2d are presented in Supplementary Figure S1. (**e**). Analysis of the Western blot in (**d**) shows that the expression of GPR19 is higher in the diabetic rats compared to control. *n* = 5–6. Scale bar: 50 μm. Independent samples *t*-test was used for data analysis. * *p* < 0.05, and ** *p* < 0.01.

The expression of the adropin peptide and GPR19 receptor in the pancreas of normal and diabetic rats was investigated using immunofluorescence staining, serum analysis, and the Western blotting technique. Adropin expression was detected in the normal and diabetic groups. However, the distribution pattern was not similar in both groups, as adropin expression in the endocrine pancreas and pancreatic acini was reported in thenormal rats, while in diabetic rats, the expression was mainly in the endocrine pancreas (Figure 2a). Also, a significant (*p* < 0.05) increase in the percentage distribution of adropin in the pancreatic endocrine of the normal rats was observed compared to the diabetic group (Figure 2b and Figure S1). Further, we measured the adropin concentration in the serum of normal and diabetic rats, and we reported a decrease (*p* < 0.05) in the adropin level in the diabetic group compared to the normal rats (Figure 2c).

Moreover, we found that pancreas tissue in both normal and diabetic rats was expressing GPR19 receptors (Figure 2d). However, the receptor expression in the diabetic group was significantly (*p* < 0.01) higher in comparison to the nondiabetic group.

### 2.2. Adropin Treatment Enhanced Glucose Tolerance in Diabetic Rats

**Figure 3:**
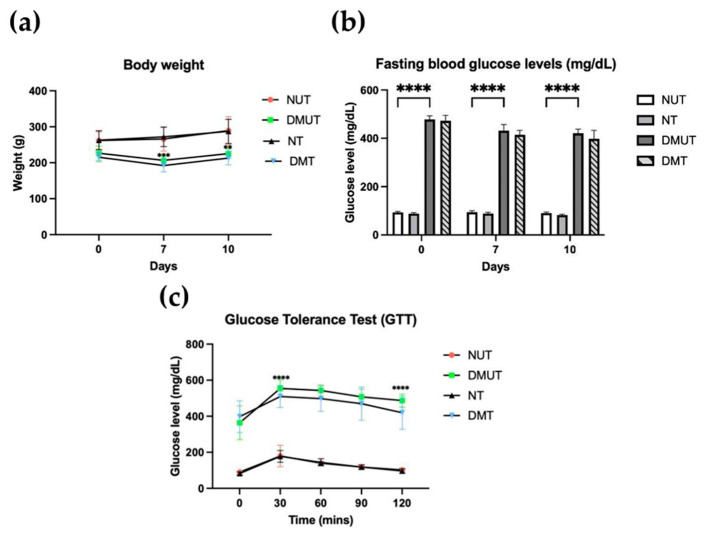
Effects of adropin on body weight and fasting blood glucose levels in control and diabetic rats. Body weight (**a**), fasting blood glucose (**b**), and GTT (**c**). Body weight significantly dropped as diabetes was induced. Blood glucose increased in diabetic rats compared to nondiabetic. *n* = 6. ANOVA test was used for data analysis. ** *p* < 0.01, *** *p* < 0.001, and **** *p* < 0.0001.

To study the effect of adropin on body weight and fasting blood glucose, body weight and fasting blood glucose were recorded, and GTT was performed (Figure 3). Body weight was significantly decreased (*p* < 0.001) in the diabetic rats compared to the nondiabetic controls. However, adropin peptide did not change the body weight in the diabetic group after treatment (Figure 3a). Fasting blood glucose increased in the diabetic group compared to the normal group (*p* < 0.0001). However, there was no different between DMUT rats and DMT rats (Figure 3a,b). For GTT, the rats received a glucose injection intraperitoneally, and blood glucose was monitored every 30 min for 2 h. All the groups experienced an increase in blood glucose 30 min after glucose injection. Interestingly, the diabetic rats treated with adropin had blood glucose levels lower than the diabetic untreated group, speculating that adropin treatment improved glucose tolerance in diabetic rats (Figure 3c).

### 2.3. Distribution of Adropin in Pancreatic Endocrine Cells and Its Effect on Pancreatic Peptide Secretion

We investigated the expression of adropin in the pancreatic endocrine cells (β, α, PP, and D cells) using immunofluorescence staining, and serum analysis was conducted to measure the endocrine hormones’ secretion using MAGPIX technology.

#### 2.3.1. Pancreatic β-Cell Expression of Adropin and Its Effect on Insulin Secretion

**Figure 4:**
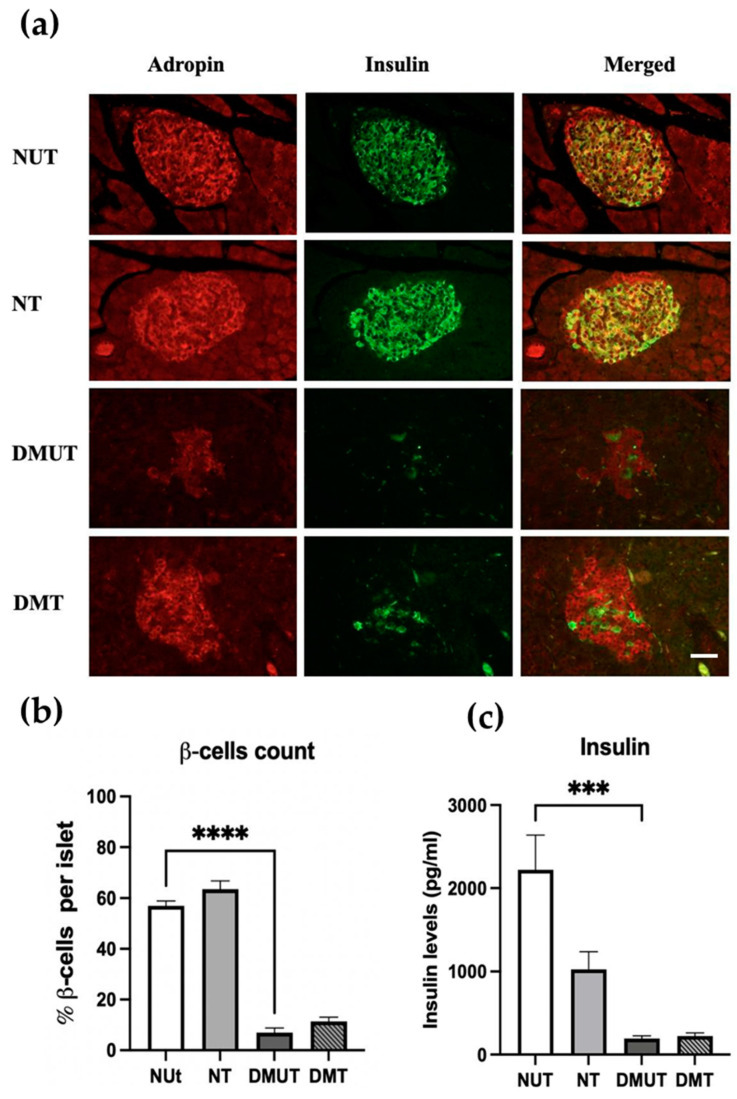
Expression of adropin in pancreatic β-cells of nondiabetic and diabetic rats (**a**) and serum insulin analysis (**c**). Colocalization of anti-adropin and anti-insulin antibodies in β-cells in both diabetic and nondiabetic rats (**a**). No change in β-cell count (**b**) or serum insulin levels between diabetic and nondiabetic rats treated with adropin. *n* = 6. Scale bar: 50 μm. ANOVA test was used for data analysis. *** *p* < 0.001, and **** *p* < 0.0001.

Double-labeling immunofluorescence for the pancreas tissue showed colocalization of anti-adropin antibody and anti-insulin antibody in both the normal controls and the diabetic rats, illustrating that pancreatic α-cells express adropin peptide (Figure 4a). In the islet of Langerhans, insulin-labeled β-cells were counted to study the effect of adropin treatment on the number of β-cells in nondiabetic and diabetic rats. We reported a significant decrease in the number of β-cells in normal controls compared to the diabetic untreated group (Figure 4b), and this was expected since β-cells are destroyed because of diabetes mellitus. However, there was no change in the number of β-cells between DMUT and DMT groups of rats. This result was in agreement with the serum analysis of insulin, as it was noticed that both nondiabetic and diabetic treated rats had almost equivalent insulin levels in serum (Figure 4c), whereas a decrease in insulin levels was reported in the untreated controls compared to the diabetic untreated group (Figure 4c, Table 1).

#### 2.3.2. Pancreatic α-Cell Expression of Adropin and Its Effect on Glucagon Secretion

**Figure 5:**
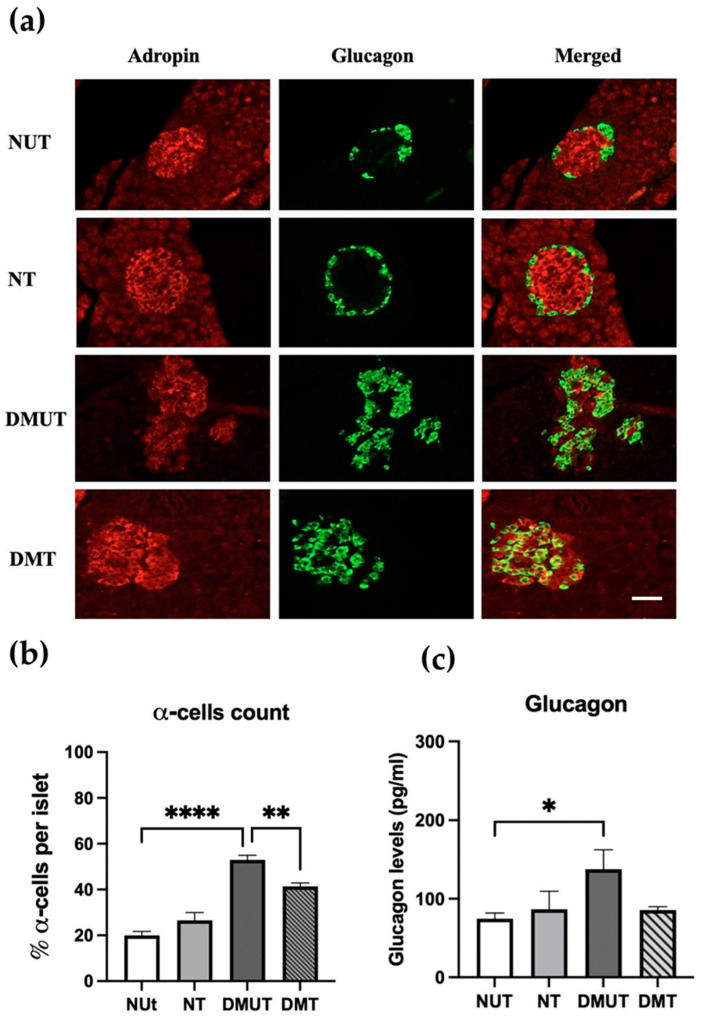
Expression of adropin in pancreatic α-cells of nondiabetic and diabetic rats (**a**) and serum glucagon analysis (**c**). Colocalization of anti-adropin and anti-glucagon antibodies in α-cells in both diabetic and nondiabetic rats (**a**). Adropin treatment decreased the α-cell number (**b**) and serum glucagon levels (**c**) in the diabetic compared to nondiabetic rats. *n* = 6. Scale bar: 50 μm. ANOVA test was used for data analysis. * *p* < 0.05, ** *p* < 0.01, and **** *p* < 0.0001.

The distribution of adropin in pancreatic α-cells in normal controls and diabetic rats was studied. Only a few cells in the islet of the normal controls showed colocalization between anti-adropin antibody and anti-glucagon antibody. However, this pattern of distribution of adropin in α-cells changed after induction of diabetes mellitus, in a way that more α-cells were expressing adropin in the islet of the diabetic rats compared to the normal controls (Figure 5a). Interestingly, the number of glucagon-producing cells significantly decreased (*p* < 0.01) in DMT compared to DMUT as adropin treatment was administered (Figure 5b).

We also investigated the effect of adropin on glucagon secretion, so serum glucagon levels were measured in all the groups. The glucagon level was significantly (*p* < 0.05) higher in the diabetic untreated rats compared to the normal control group. With adropin treatment, glucagon secretion from α-cells decreased in the DM treated compared to the diabetic untreated rats (Figure 5c, Table 1).

#### 2.3.3. Pancreatic PP-Cell Expression of Adropin and Its Effect on Pancreatic Polypeptide Secretion

**Figure 6:**
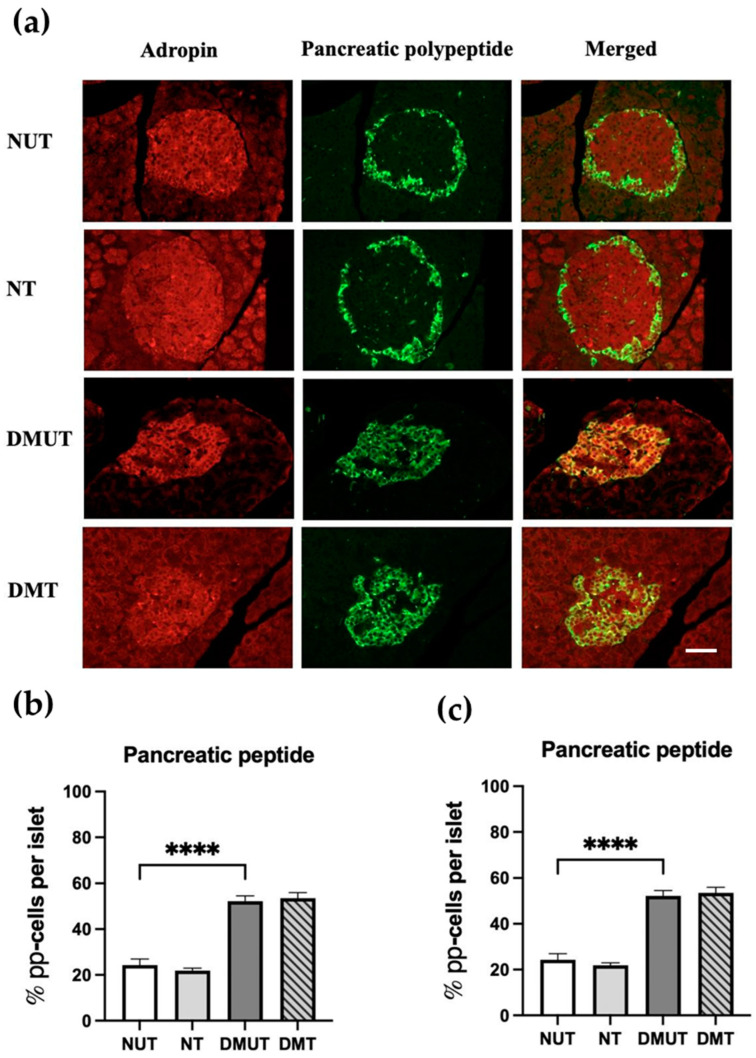
Expression of adropin in pancreatic polypeptide cells of nondiabetic and diabetic rats (**a**) and serum pancreatic polypeptide hormone analysis (**c**). Colocalization of anti-adropin and anti-PP antibodies in PP-cells in both diabetic and nondiabetic rats (**a**). Adropin treatment increased the PP-cell number (**b**) and serum pancreatic polypeptide hormone levels (**c**) in the diabetic compared to nondiabetic rats. *n* = 6. Scale bar: 50 μm. ANOVA test was used for data analysis. **** *p* < 0.0001.

The histological analysis showed colocalization between anti-adropin and anti-pancreatic polypeptide antibodies in both normal controls and diabetic rats (Figure 6a). By counting anti-pancreatic polypeptide-labeled cells, there was an increase in the percentage of PP-cells as diabetes was induced. However, the PP-cell count in the diabetic rats remained the same when adropin treatment was performed (Figure 6b). Moreover, an increment in pancreatic polypeptide hormone release to the serum was reported in both diabetic treated and untreated groups (Figure 6c).

#### 2.3.4. Pancreatic D-Cell Expression of Adropin

**Figure 7:**
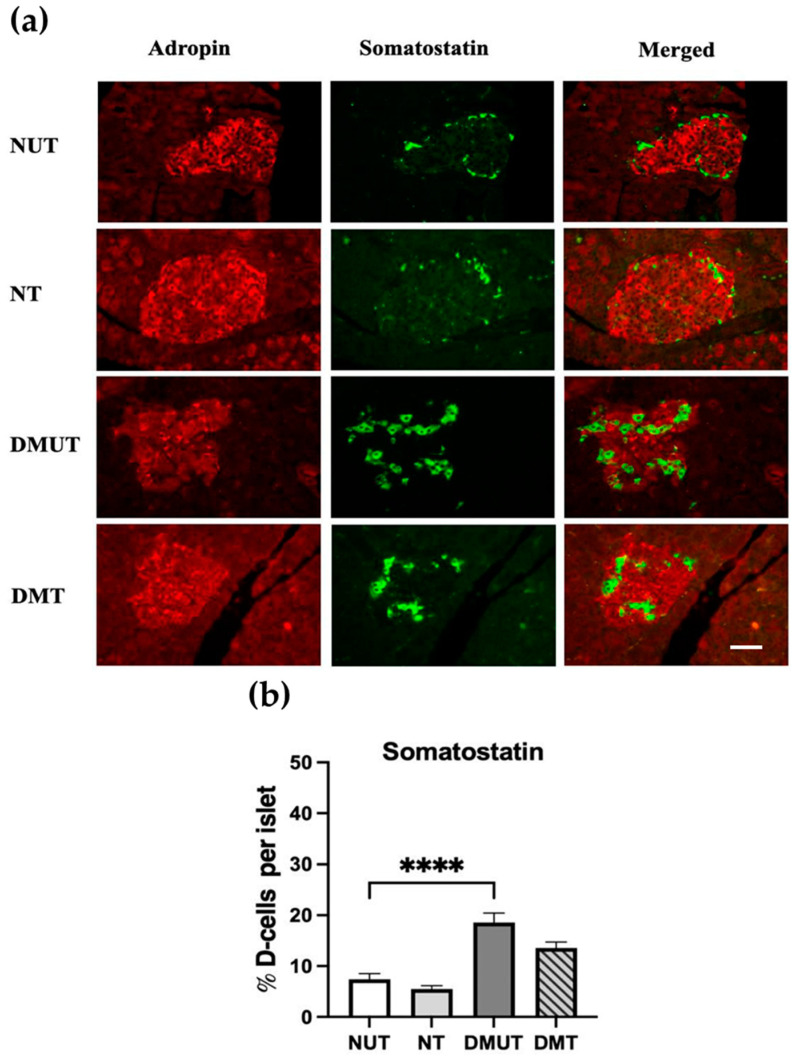
Expression of adropin in pancreatic D-cells of nondiabetic and diabetic rats. Colocalization of anti-adropin and anti-somatostatin antibodies in D-cells in both diabetic and nondiabetic rats (**a**). Adropin treatment increased the D-cell number in the diabetic rats compared to nondiabetic rats (**b**). *n* = 6. Scale bar: 50 μm. ANOVA test was used for data analysis. **** *p* < 0.0001.

We investigated the expression of adropin in somatostatin-producing D-cells. Double-labeling immunofluorescence for the pancreas tissue showed colocalization of anti-adropin antibody and anti-somatostatin antibody in both the normal controls and the diabetic rats, illustrating that pancreatic D-cells express adropin peptide (Figure 7a). Islets of the diabetic rats showed a considerably higher number of D-cells than the normal controls (Figure 7b). The effect of adropin treatment on the pancreatic D-cell number was further investigated, and no evidence of alterations in cell number was found in both diabetic and nondiabetic rats after treatment (Figure 7b, Table 1).

### 2.4. Distribution of Adropin in the Cytoplasmic Granules of β- and α-Cells

**Figure 8:**
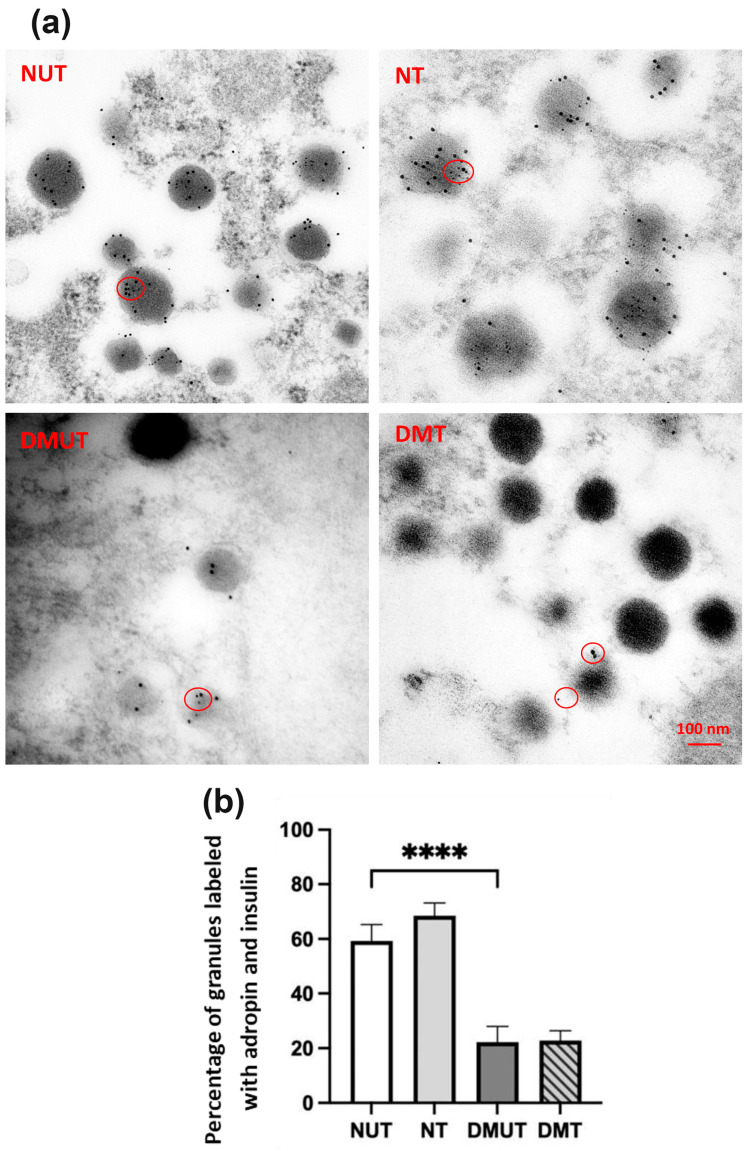
Immunoelectron microscopy of pancreatic β-cells in nondiabetic and diabetic rats. Colocalization of adropin-labeled immunogold particles (6 nm) with insulin-labeled immunogold particles (12 nm) at the intracellular level is shown as red circles (**a**). DMUT had a lower number of granules with colocalized adropin and insulin than NUT (**b**). *n* = 6. Scale bar: 100 nm. ANOVA test was used for data analysis. **** *p* < 0.0001.

**Figure 9:**
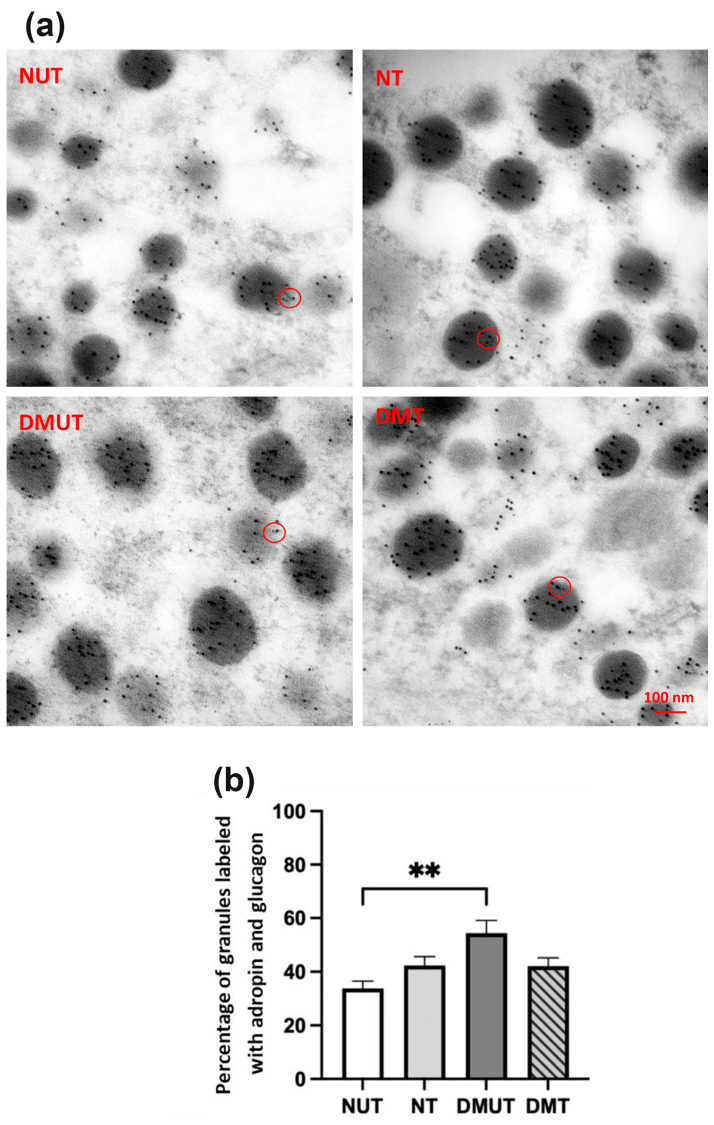
Immunoelectron microscopy of pancreatic α-cells in nondiabetic and diabetic rats. Colocalization of adropin-labeled immunogold particles (6 nm) with glucagon-labeled immunogold particles (12 nm) at the intracellular level is shown as red circles (**a**). DMUT had a higher number of granules with colocalized adropin and glucagon than NUT (**b**). *n* = 6. Scale bar: 100 nm. ANOVA test was used for data analysis. ** *p* < 0.01.

Immunoelectron microscopy was used to detect the colocalization of adropin-labeled immunogold particles. General observations were that nondiabetic rats had a higher number of insulin secretory granules in pancreatic β-cells compared to the diabetic animals. Insulin granules appeared in gray color surrounded by a large halo, and more empty vesicles were observed in the diabetic group (Figure 8, Table 1). In pancreatic α-cells, glucagon granules were darker in color than insulin granules and more abundant in diabetic rats than in the normal control group (Figure 9, Table 1).

Insulin secretory granules in β-cells contained both adropin-labeled immunogold particles and insulin-labeled immunogold particles (Figure 8a), which confirmed our earlier finding in IHC. The colocalization of adropin and insulin in the cytoplasmic organelles of β-cells significantly (*p* < 0.0001) dropped in the diabetic rats compared to normal controls (Figure 8b). On the other hand, no evidence of an increase in the percentage of granules with both adropin and insulin was reported in the diabetic group as adropin treatment was performed.

Adropin-labeled immunogold particles were also found co-existing with glucagon-labeled immunogold particles in the glucagon secretory granules of pancreatic α-cells (Figure 9a). The intracellular granule percentage with both adropin and glucagon labeling was found significantly (*p* < 0.01) higher in diabetic rats compared to normal controls. Adropin treatment slightly decreased the colocalization of these two peptides in the granules (Figure 9b).

### 2.5. Peptide Hormone Analysis

**Figure 10:**
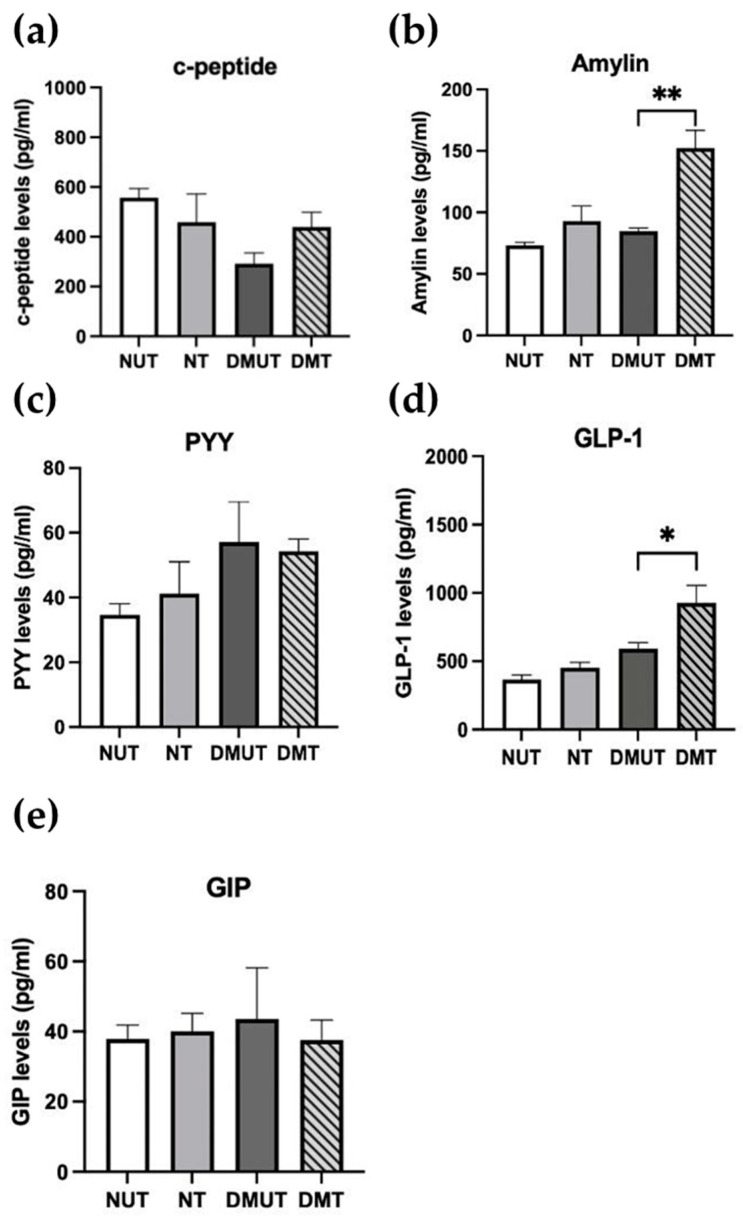
Effect of adropin on pancreatic hormones and hormones involved in glucose metabolism in diabetic rats. Adropin slightly but not significantly increased serum levels of c-peptide (**a**), amylin (**b**), and GLP-1 (**d**) in the diabetic rats, while there was no change in PYY (**c**) and GIP (**e**). *n* = 4–6. ANOVA test was used for data analysis. * *p* < 0.05, ** *p* < 0.01.

We investigated how adropin could affect the secretion of some endocrine pancreatic hormones, such as c-peptide, amylin, and PYY, as well as other hormones involved in glucose metabolism, such as GLP-1 and GIP, using the MILLIPLEX^®^ MAP instrument (Milliplex, Billerica, MA, USA; Figure 10, Table 1).

The serum c-peptide level was lower in the diabetic rats compared to the normal controls; however, adropin administration slightly stimulated c-peptide secretion in DMT (Figure 10a). There was no difference in amylin serum levels between diabetic rats and the normal controls. Interestingly, amylin secretion significantly (*p* < 0.01) increased in the diabetic group treated with adropin (Figure 10b). Induction of diabetes caused a slight rise in PYY serum levels compared to the nondiabetic animals. However, no alteration was reported as adropin treatment was conducted (Figure 10c).

The normal control rats had slightly lower GLP-1 serum levels than the DMUT group. Interestingly, adropin augmented GLP-1 serum levels significantly (*p* < 0.05) in the diabetic group (Figure 10d). On the other hand, there was no variation in GIP serum levels among the diabetic and nondiabetic rats, and this remained the same with adropin treatment (Figure 10e). GLP-1 and GIP are incretins that induce insulin secretion from β-cells.

### 2.6. Insulin and Glucagon Secretion from Pancreatic Tissue Fragments and INS-1 832/3 Rat Insulinoma Cell Line

**Figure 11:**
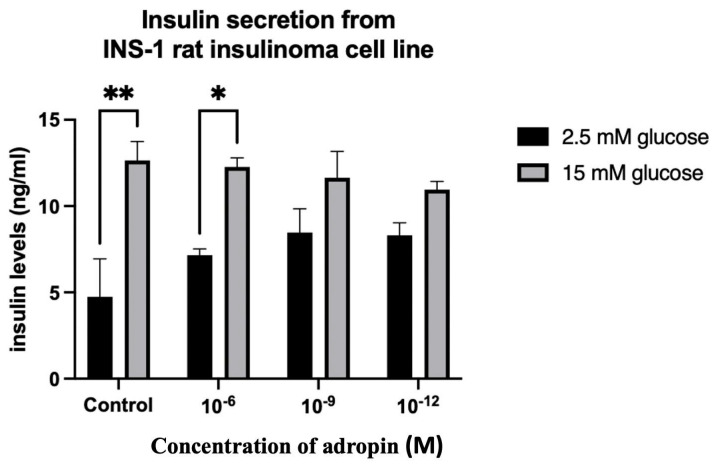
Effect of adropin on glucose stimulation of insulin secretion from the INS-1 832/3 rat insulinoma cell line. Here, 15 mM of glucose caused higher insulin release compared to 2.5 mM. These increases in insulin release were not statistically different within groups. Adropin had no effect on insulin secretion from the β-cell line. ANOVA test was used for data analysis. * *p* < 0.05, ** *p* < 0.01.

In the INS-1 832/3 rat insulinoma cell line, the cells were stimulated to release insulin by performing low- and high-glucose concentration incubation (2.5 mM and 15 mM, respectively). Generally, 15 mM glucose-stimulated insulin released more than 2.5 mM of glucose (Figure 11, Table 1). Adropin did not change the insulin release from the β-cell line stimulated with 2.5 mM and 15 mM of glucose.

**Figure 12:**
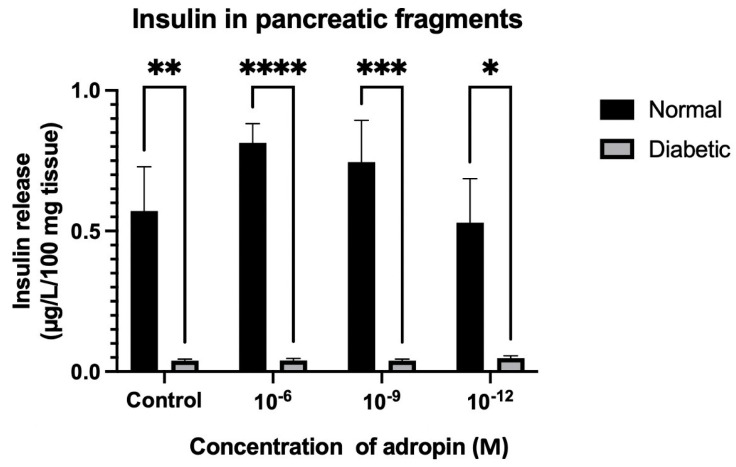
Effect of adropin on insulin release from pancreatic tissue fragments of normal and diabetic rats. Insulin secretion from pancreatic tissue fragments of diabetic rats was significantly lower than the normal group. Adropin did not stimulate insulin release from pancreatic tissue fragments of diabetic rats. *n* = 6. ANOVA test was used for data analysis. * *p* < 0.05, ** *p* < 0.01, *** *p* < 0.001, and **** *p* < 0.0001.

**Figure 13:**
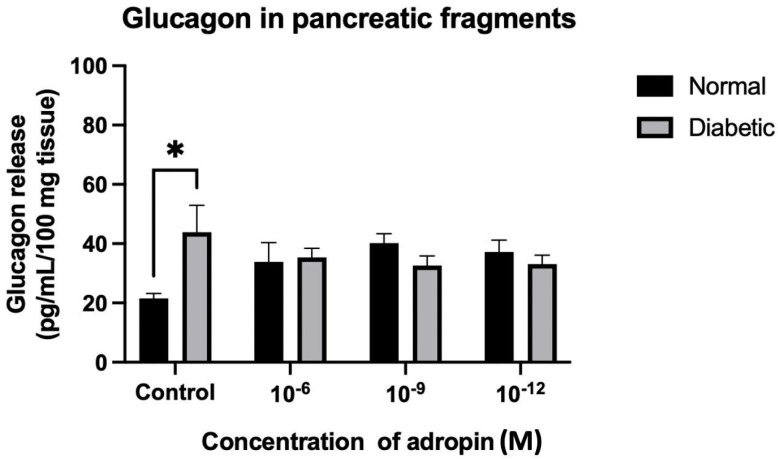
Effect of adropin on glucagon release from pancreatic tissue fragments of normal and diabetic rats. Glucagon secretion from pancreatic tissue fragments of diabetic rats was significantly higher than the normal group with no peptide treatment. Pancreatic tissue fragments of diabetic rats secreted glucagon in similar levels as the normal group with adropin treatment. *n* = 6. ANOVA test was used for data analysis. * *p* < 0.05.

In the pancreatic tissue fragments, insulin was significantly (*p* < 0.01) lower in the diabetic rats compared to normal rats in the control group with no peptides. As the tissue fragments were treated with adropin, the diabetic group remained significantly lower than the normal rats (Figure 12, Table 1). There was no variation in insulin release stimulation detected among the diabetic groups treated with different concentrations of adropin (Figure 12). On the other hand, stimulation of glucagon release from pancreatic tissue fragments with different concentrations of adropin (10^−6^, 10^−9^, and 10^−12^ M) resulted in an equivalent amount of glucagon release in the normal and diabetic rats, but not in the groups with no peptide treatment (Figure 13). A significant (*p* < 0.05) increase in glucagon release was reported from tissue fragments of the diabetic rats, in comparison to the normal rats with no peptide stimulation. Adropin demolished the difference in glucagon secretion between normal and diabetic rats, as both had similar glucagon levels (Figure 13). From this finding, we could say that adropin may affect glucagon release in diabetes mellitus.

## 3. Discussion

### 3.1. Adropin and GPR19 Are Expressed in Pancreas of Normal and Diabetic Rats

The first discovery of adropin, by Kumar et al., illustrated the expression of *Enho* mRNA, the gene encoding for adropin peptide, in the liver and brain of human and lean B6 mice [10]. Moreover, tissue expression of adropin was shown in the liver, brain, kidney, lung, and pancreas [13]. In our study, we demonstrated adropin expression in the pancreas of normal and STZ-induced diabetic rats. The results showed the distribution of adropin in the pancreatic islet in both normal and diabetic rats; however, the expression was significantly lower in the pancreatic islet of the diabetic compared to normal rats. On the other hand, adropin was detected only in pancreatic acinar cells of the normal rats, but not in the diabetic rats. Using the immunohistochemical technique, other studies confirmed the expression of adropin in acinar cells and islet capillaries of the pancreas [13,14]. Serum adropin levels were also investigated. We reported a significant decrease in serum adropin levels after inducing diabetes in comparison to the normal rats. It is worth mentioning that a decline in serum adropin was observed in the Chinese population with T2DM [18] as well as in type 1 diabetic children [19].

Moreover, we investigated the expression of the GPR19 receptor in the pancreas of normal and diabetic rats. Using the Western blotting technique, we reported expression of GPR19 in both normal and diabetic rats’ pancreas; however, it was relatively higher in the diabetic group. Expression of the *GPR19* gene was detected earlier in isolated pancreatic islets of rats [27] as well as the alpha TC1-9 pancreatic alpha cell line [28].

GPR19 is an orphan receptor, and adropin was identified as one of its ligands [11]. It is abundantly found in the brain and expressed in other organs, such as the liver and heart [29,30]. GPR19 was recently found expressed in the suprachiasmatic nucleus of the hypothalamus, the main part of the brain for the circadian clock, suggesting GPR19′s potential role in regulating circadian rhythm [30]. Furthermore, overexpression of GPR19 in breast cancer cells was linked to regaining the tumor cells to epithelial characteristics by expressing E-cadherin, which facilitates metastatic colonization of the tumor [31]. A recent study did not confirm that GPR19 is an adropin receptor [32].

### 3.2. Adropin Enhanced Glucose Tolerance in Diabetic Rats, but Did Not Change Body Weight and Fasting Blood Glucose

Body weight for the normal and diabetic rats was recorded over the period of the experiment (four weeks). Prior to inducing diabetes, all the groups had almost the same weight. There was a significant drop in body weight for the diabetic rats after the onset of diabetes. Several studies reported weight loss in animals with STZ-induced diabetes compared to normal controls [33,34]. Losing body weight after induction of diabetes is probably due to the destruction of insulin-producing β-cells by STZ drugs and insulin deficiency. In insulin-dependent diabetic patients, lack of insulin hinders the body from delivering blood glucose to the body’s cells for use as fuel. When this happens, the body begins utilizing fat and muscle for energy, which results in a loss of total body weight. Further, adropin treatment did not change the body weight in normal and diabetic untreated and treated rat groups over the 10 days of administration. In agreement with our results, in another study, DIO mice treated with five intraperitoneal injections of adropin over 2–3 days did not experience weight alteration, in comparison to DIO mice that received vehicle [26].

Furthermore, fasting blood glucose increased in STZ-induced diabetic rats compared to normal controls. The defect in insulin secretion caused insulin-sensitive tissues to be unable to respond and uptake glucose from the bloodstream, leading to elevations in blood glucose. When adropin was administered to the nondiabetic and diabetic rats, fasting blood glucose levels remained the same compared to untreated groups. Thus, adropin did not change fasting blood glucose levels in diabetic treated rats in comparison to diabetic untreated rats, as we indicated earlier in this study. In contrast to this result, Thapa et al. reported a decrease in fasting blood glucose levels in mice fed a high-fat diet after adropin administration, compared to mice fed a low-fat diet [23]. It was suggested that adropin reduces hepatic glucose production via downregulation of gluconeogenic enzymes, such as glucose 6-phosphatase [23].

Moreover, we examined the effect of adropin peptide on glucose tolerance in diabetic rats by administering a single injection of glucose intraperitoneally and then recording blood glucose levels for 2 h at every 30 min interval. Interestingly, our result indicated enhanced glucose tolerance in adropin-treated diabetic rats, compared to the diabetic untreated rats. The ability of adropin to improve glucose tolerance has been reported in several studies that used DIO mice [23,26]. These findings collectively imply that administering adropin peptide to diabetic rats and DIO mice was successful in improving glucose tolerance. Gao et al. suggested that in case of obesity, adropin may alter fuel utilization in muscle from fatty acid oxidation toward glucose oxidation and utilization, which may enhance whole-body glucose tolerance [26].

### 3.3. Adropin Distribution in Pancreatic Islet Cells and Its Effect on Peptide Secretion

Earlier in this study, we showed adropin expression in the exocrine and endocrine pancreas of normal and diabetic rats. Then, we investigated the cellular distribution of adropin in the endocrine pancreas of normal and diabetic rats. The double-labeling immunofluorescence technique was performed using specific antibodies against adropin, insulin, glucagon, somatostatin, and pancreatic polypeptide. Moreover, we measured pancreatic endocrine peptide secretion in the serum of normal and diabetic rats treated with adropin using MAGPIX technology.

Insulin-positive β-cells were detected abundantly in the center of the islet of Langerhans of the normal rats, while in islets of the diabetic rats, insulin-positive β-cells were found very few to none in number, and this was due to diabetes induction and β-cell destruction using STZ. The same pancreatic β-cell pattern of distribution in both normal and diabetic rats was illustrated in [35,36]. Further, we showed the colocalization of anti-adropin and anti-insulin antibodies in β-cells of the normal and diabetic rats treated and untreated groups, demonstrating the expression of adropin in pancreatic β-cells. Also, considering the reduction in β-cell number in the diabetic untreated group, adropin administration changed neither the β-cell count nor the serum insulin level in the diabetic-treated rats. It is well known that in T1DM, the overall β-cell mass is dramatically reduced, and the serum insulin level drops as well [8,37]. It is worth mentioning that some peptides, such as GLP-1, have an anti-diabetic effect by enhancing insulin release from β-cells [38]; however, based on our results, adropin cannot restore β-cells nor stimulate insulin secretion. Adropin expression in pancreatic α-cells was investigated. In the normal control rats, glucagon-positive α-cells were localized peripherally in the islet, while in diabetes-induced rats, glucagon-positive α-cells were abundantly found in the central as well as peripheral regions of the islet. Similar distributions of pancreatic α-cells in both normal and diabetic rats were reported earlier [35,36]. Colocalization of adropin and glucagon was detected in the normal and the diabetic animals, suggesting expression of adropin in α-cells. Serum glucagon levels were found to be markedly elevated in the diabetic untreated rats, which was due to the significant increase in the number of glucagon-secreting α-cells compared to the normal controls. With adropin treatment, the percentage of α-cells per islet was reduced, and the serum glucagon level was decreased in the diabetic treated animals, in comparison to diabetic untreated. The physiological function of glucagon is elevating blood glucose during hypoglycemia by stimulating hepatic glucose production and inhibiting glycogen synthesis. However, in diabetes mellitus, there is dramatic excess of glucagon release by α-cells, attributed to β-cell dysfunction, as well as a lack of suppression of glucagon secretion, thus contributing to increased hepatic glucose production, consequently causing hyperglycemia [5]. Another defect of α-cells is that in T1DM and long-lasting T2DM, α-cells do not respond to low glucose levels, raising the risk of having severe hypoglycemic episodes, especially among insulin-receiving patients [39]. The abnormal function of α-cells in diabetes is mainly caused by impairment of insulin release or action leading to failure of glycemic control [5]. Adropin expression was detected in the pancreatic-polypeptide-secreting PP-cells of normal and diabetic rats. PP-cells are located at the periphery of the islet of Langerhans in normal conditions [35]; however, in diabetes mellitus, the histological distribution of PP-cells changes to dominate the central region of the islet [40]. Our data also reported an increase in the percentage of PP-cells in the islet of diabetic untreated animals compared to the normal controls, while the PP-cell count remained the same in the diabetic animals, after adropin administration. A similar result was observed in the serum analysis for pancreatic polypeptide secretion that we conducted. Pancreatic polypeptide is a pancreatic endocrine as well as gastrointestinal hormone, mainly secreted after protein intake. It is believed that pancreatic polypeptide plays a role in insulin and glucagon secretion through a paracrine relationship [41]. Evidence showed that pancreatic polypeptide is involved in regulating the insulin receptor gene in the liver, consequently enhancing insulin sensitivity [42]. Moreover, Zhao et al. demonstrated that impaired glucose tolerance is associated with increased release of pancreatic polypeptide postprandially in patients with T2DM [43]. In our study, adropin could not alter pancreatic polypeptide secretion in the serum of diabetic treated rats.

Only limited cells were found positive for somatostatin in the islet of nondiabetic rats. The somatostatin-labeled D-cell number increased significantly as diabetes was induced. Colocalization of adropin- and somatostatin-labeled cells was seen in both diabetic and normal animals. However, adropin treatment did not change the percentages of D-cell numbers in islets of STZ-induced diabetic rats. In our study, we observed suppression of glucagon secretion in diabetic rats when adropin was administered; however, neither suppression of pancreatic polypeptide secretion nor enhancement of insulin release was detected. Based on the obtained results, it can be assumed that the effect of adropin on the pancreas is on α-cells, so it is important to understand what regulates glucagon secretion and if adropin is involved in these mechanisms. Other than circulating glucose levels, which is the main trigger that modulates glucagon release, glucagon secretion is regulated by several factors, including hormones, circulating nutrients, and neuronal transmitters. Insulin and somatostatin regulate glucagon secretion through paracrine control. Insulin regulation to glucagon secretion is one of the key paracrine mechanisms. Several pathways are involved in inhibition of glucagon secretion by insulin, such as the insulin receptor and phosphatidylinositol 3-kinase (PI3K) signaling pathway. Insulin receptors are expressed on α-cells, and once the receptor is activated, K_ATP_ channel activities are increased through the PI3K signaling pathway, causing membrane hyperpolarization and glucagon suppression [44]. Moreover, it has been reported that Zn^2+^ atoms released during insulin hexamer dissociation can modulate glucagon secretion by altering K_ATP_ channel activities [44]. Another paracrine regulation of glucagon secretion is through somatostatin secretion from D-cells. Somatostatin inhibits glucagon secretion via binding to somatostatin receptors on α-cells and then inhibiting glucagon granules’ exocytosis [45]. GLP-1 also suppresses glucagon secretion by depolarizing α-cell membrane potential and decreasing the action potential by acting on GLP-1 receptors on α-cells [46]. Besides hormones, nutrients, such as fatty acids and amino acids, can regulate glucagon release. Different amino acids have distinct effects on α-cells and glucagon release. Amino acids, such as arginine, alanine, and glutamine, trigger glucagon secretion, while isoleucine and lysine amino acids were reported to work as negative modulators for glucagon release [47]. Since amino acids also promote the release of insulin, it is hypothesized that the purpose of the increase in glucagon release is to physiologically prevent hypoglycemia following protein consumption. Fatty acids have been also shown to modulate glucagon secretion in α-cells. It is worth mentioning that the effect of fatty acids on pancreatic α-cells depends on the length of the fatty acid and the duration of treatment. For instance, short-term treatment of palmitate can induce glucagon release, while chronic exposure can inhibit the secretion of glucagon [47]. In diabetes mellitus, free fatty acids are involved in the pathogenesis of the disease, and the chronic increase in fatty acids might contribute to α-cell dysregulation, specifically in T2DM.

Since we did not report any changes in insulin and somatostatin levels with adropin treatment, most probably adropin did not inhibit glucagon release through paracrine regulation on insulin and somatostatin. In fact, Billert et al. showed that adropin administration did not stimulate insulin secretion from rat pancreatic islets in the presence of 2.8 mmol/L of glucose; however, insulin release was suppressed by adropin at a high glucose concentration (16.7 mmol/L) [27]. Thus, the effect of adropin on α-cells could be a direct effect through the receptor, as Rossiter et al. reported that alpha TC1-9 (α-cell line) expresses GPR19 [28]. Another suggested mechanism is the effect of GLP-1 on glucagon secretion, as we reported a significant increase in GLP-1 serum levels in the DMT group comparing to DMUT.

### 3.4. Immunoelectron Microscopy Study of Adropin in Pancreatic Islet Cells

To determine the intracellular localization of adropin in the pancreatic islet cells, immunoelectron microscopy was performed. Insulin-containing granules in β-cells and glucagon-containing granules in α-cells were determined. Insulin granules are morphologically characterized by an electron-dense core and clear halo [36,48]. In pancreatic α-cells, glucagon granules are electron-dense without a clear halo, and they are darker in color compared to insulin granules [48]. Localization of adropin in the secretory granules of insulin and glucagon was observed in the pancreas of normal and diabetic rats. Our immunofluorescence results showed a significant decrease in the percentage of α-cells in adropin-treated diabetic rats, and our immunoelectron microscopy results also revealed a decrease in the percentage of granules containing adropin and glucagon in that same animal group, suggesting the ability of adropin to regulate glucagon secretion in diabetes.

### 3.5. Effect of Adropin Treatment on Some Peptide Hormones’ Secretion in Normal and Diabetic Rats

Other than insulin, glucagon, and pancreatic polypeptides, we also measured serum levels of other peptides involved in glucose homeostasis, such as amylin, c-peptide, PYY, GLP-1, and GIP. Using MAGPIX technology, we quantified the serum levels of these peptides in adropin-treated diabetic and nondiabetic rats.

C-peptide and amylin are both secreted from pancreatic β-cells, along with insulin. C-peptide, which stands for connecting peptide, is a product of proinsulin cleavage to yield mature insulin and free c-peptide. Interestingly, both c-peptide and insulin co-exist in the secretory granules of β-cells in equal ratios, and both are secreted together when the cell is stimulated by hyperglycemia [49]. Our results showed that serum c-peptide levels decreased when diabetes was induced in rats, as we also reported a significant decrease in serum insulin levels earlier. This was expected since β-cells were damaged due to STZ administration in diabetic animals, depleting insulin and c-peptide contents. However, with adropin treatment, the c-peptide serum concentration slightly increased in the diabetic treated rats, in comparison to the diabetic untreated rats. Knowing that c-peptide has a longer half-life than insulin and can be used as a reliable marker for β-cell activities [49], and that we detected an increase in serum c-peptide with adropin administration, but not insulin, we suggest that β-cell function could be restored by adropin in diabetic subjects. Another peptide that is co-secreted with insulin from β-cells is amylin. Amylin is stimulated by nutrients, such as glucose, amino acids, and fatty acids. It plays a role in reducing blood glucose levels, increasing satiety, and delaying gastric emptying [50]. Amylin can regulate glucose homeostasis by inhibiting postprandial glucagon release [51,52]. In T1DM, amylin secretion is extremely low due to β-cell destruction, and there is lack of amylin response to caloric intake. In T2DM, serum amylin levels depend on the stage of the disease. At early stages of T2DM, amylin secretion is high; however, at late stages, patients have low amylin levels [53]. In our study, we reported a significant increase in serum amylin in diabetic rats treated with adropin compared to diabetic untreated rats, suggesting a stimulatory effect of adropin for amylin secretion. Additionally, we investigated the effect of adropin on the secretion of a group of gut hormones that are involved in glucose homeostasis, including PYY, GLP-1, and GIP. As we induced diabetes into the rats, the serum concentration of PYY slightly increased, but there was no change in GLP-1 and GIP compared to the normal controls. With adropin administration, GLP-1 serum content significantly increased in diabetic animals, in comparison to diabetic untreated rats, while PYY and GIP levels remained the same. PYY and GLP are secreted from enteroendocrine cells, known as L-cells, mainly found in the distal gastrointestinal tract and stimulated by food intake, while GIP is released by enteroendocrine K-cells [54]. PYY plays a role in reducing appetite and regulating satiety in humans [55]. According to Le Roux et al., impaired satiety was linked to reduced postprandial PYY release in obese subjects [56]. Interestingly, PYY is also expressed in the pancreatic islet, and it is involved in maintaining glucose metabolism along with GLP-1 after caloric intake. An insulinotropic effect of PYY, GLP-1, and GIP was reported, as well as their capability to suppress glucagon secretion [57,58]. Since we demonstrated that adropin had no effect on PYY and GIP, but significantly increased serum GLP-1, we suggest that the effect of adropin on glucagon suppression in the diabetic treated rats could be due to GLP-1 increment and the activation of the GPL-1 receptor. In a recent study by Li et al., a link between adropin and the GLP-1 receptor was illustrated when diabetic rats were treated with myricetin, a plant-derived flavonoid and agonist for the GLP-1 receptor. It has been shown that circulating adropin was increased through GLP-1 receptor activation via myricetin [59]. So, we suggest a modulatory role of adropin in the endocrine activities of pancreatic α-cells.

### 3.6. Insulin and Glucagon Secretion from Pancreatic Tissue Fragments and INS-1 832/3 Rat Insulinoma Cell Line Treated with Adropin

We investigated the effect of adropin on insulin and glucagon secretion using pancreatic tissue fragments of normal and diabetic rats and the INS-1 832/3 rat insulinoma cell line. Our study showed that insulin release from the INS-1 832/3 rat insulinoma cell line did not change when cells were treated with different concentrations of adropin, regardless of glucose stimulation. We also observed the same finding when pancreatic tissue fragments of the diabetic rats were treated with the same concentrations of adropin. In a previous study by Billert et al., significant suppression of glucose-induced insulin release as well as insulin gene expression was reported in both INS-1E cells and pancreatic islets of normal rats treated with adropin [27]. The team demonstrated this effect of adropin through the ability of the peptide to downregulate cAMP, which is important for insulin exocytosis. In contrast to our results and Billert et al.’s findings, several studies reported the important role of adropin in enhancing insulin sensitivity and improving insulin tolerance. The concentrations of adropin used for the treatment of cell lines and tissue fragments were slightly different from that of Billert et al., albeit with a little overlap. The concentrations of 10^−6^–10^−12^ M that we used have been reported to be broad enough to cover a large range of pharmacological doses of peptide hormones [60,61,62].

Another finding in our study was the interesting modulation of glucagon secretion after adropin treatment for pancreatic tissue fragments of diabetic rats. A significant increase in glucagon was reported in pancreatic tissue fragments of the diabetic rats in comparison to the normal rats when both were treated with adropin-free Krebs solution. However, when the tissue fragments were treated with adropin, the difference was abolished, and glucagon levels in diabetic rats became equivalent to the normal rats. In a recent study, alpha TC-9 cells were treated with adropin, and the peptide had no direct effect on the expression level of proglucagon transcript. However, it is worth mentioning that adropin strongly increases the GPR183 transcript, which can directly promote proglucagon transcription through receptor activation [28]. Generally, little is known about the effect of adropin on α-cells and glucagon secretion in diabetes mellitus. More research is needed to study the anti-diabetic effect of adropin via α-cell modulation.

Beyond peptide therapy, there are several significant and emerging topics in the approach to diabetes mellitus. These include a focus on key areas of research and clinical practice, such as the role of gut microbiota modifications in insulin production and glycemic control [63,64] and research into the genetic basis of diabetes by allowing for more predictive analytics and precision medicine strategies [65].

## 4. Materials and Methods

### 4.1. Animals and Experiment Design

This study was approved by the Ethics committee of the United Arab Emirates University (UAEU), approval number A5-14, and dated 8 April 2014. Male Wistar rats, weighting between 200 and 250 g, were used in this study. The rats were obtained from the Animal Facility at the College of Medicine and Health Sciences, United Arab Emirates University, and maintained in a Specific Pathogen Free (SPF) environment, at approximately 25 °C with a 12 h light/dark cycle. The animals (*n* = 24) were fed normal chow with free access to water. The 3Rs principle in animal research was followed to minimize the number of rats required by using in vitro methods and applying statistical methods to ensure the minimum number of rats in use, while still achieving scientifically valid results. To minimize pain and distress, the least invasive method of sacrifice was implemented, and appropriate anesthesia was used. First, the rats were randomly divided into two groups, the normal group (*n* = 12) and the to-be-diabetic group (*n* = 12). For diabetes mellitus induction, the rats received a single injection of 60 mg/kg of streptozotocin (STZ; Sigma Life Science, Burlington, MA, USA) intraperitoneally, and only animals with fasting blood glucose >250 mg/dL were considered diabetic. For adropin treatment, after 2 weeks from diabetes induction, the normal and diabetic rats were randomly subdivided, as detailed in Table 2. For 10 days, the treated groups received adropin peptide (Phoenix Pharmaceuticals, Burlingame, CA, USA) at a dose of 2.1 μg/kg/day intraperitoneally. This dose has been verified in the literature [66].

Body weight and fasting blood glucose were recorded on day 0, day 7, and day 10. Blood samples from the tail vein of the rat were used to measure blood glucose levels by a OneTouch^®^ Ultra^®^ glucometer (LifeScan, Malvern, PA, USA). A glucose tolerance test (GTT) was conducted by administering the rats a glucose injection (2 g/kg) intraperitoneally. Blood glucose levels were recorded every 30 min for 2 h.

### 4.2. Blood and Tissue Collection

The rats were anesthetized using a xylazine (5 mg/kg) and ketamine (75 mg/kg) cocktail and then sacrificed. Blood was collected immediately after sacrifice from the inferior vena cava and placed in a BD vacutainer. The tubes were centrifuged at 906 rcf for 15 min for serum separation. The supernatant was collected (serum) in a new Eppendorf tube and stored at −80 °C for the subsequent analysis. Pancreas was collected from all the groups. The tissue was divided into 2 parts. One part was kept in Zamboni fixative for immunofluorescence staining and the second part was kept in McDowell fixative for the immunoelectron microscopy technique.

### 4.3. Histological Analysis

For light microscopy examination, the collected pancreas tissues were fixed in Zamboni fixative for 48 h, washed with phosphate-buffered saline (PBS), and then stored in 70% ethanol. After that, the tissues were dehydrated using series of ascending concentrations of ethanol (70%, 95%, and 100%) and then infiltrated in paraffin wax. Following that, the processed tissues were paraffin wax embedded to make paraffin-embedded tissue blocks. The tissue blocks were sectioned (5 μm) using a microtome, and each section was placed on a slide for further staining [67].

For electron microscopy examination, pancreas tissues were kept in McDowell fixative for 48 h, washed with PBS, and then stored in 70% ethanol. After that, the tissues were trimmed to around 4 mm^3^ and dehydrated using a series of ascending concentrations of ethanol (70% and 95%). LR white resin was used for infiltration and embedding. After that, the polymerization step was performed to make tissue blocks by placing the pancreas tissue in gelatin capsules with pure LR white for adequate time under UV lamp irradiation with a wavelength of 360–365 nm. Then, the blocks were trimmed and sectioned. Ultra-thin sections were obtained using an ultramicrotome, and the tissue sections were placed on copper grids for immunoelectron microscopy staining [68].

#### 4.3.1. Immunofluorescence Staining of Paraffin Sections

After paraffin wax embedding and tissue sectioning, the sections were incubated in a 60 °C incubator for 10 min to remove the wax from the tissue. Then, the sections were kept in xylene I and II, each for 5 min. After that, the sections were rehydrated using series of descending concentrations of ethanol (100%, 95%, 70%, and 50%, respectively) and then distilled water for 5 min. After that, antigen retrieval was performed using sodium citrate buffer (10 mM sodium citrate, 0.05% Tween 20, pH 6.0): The slides were kept in citrate buffer and placed in a microwave, and the temperature was set as high-power P10 for 2 min, then low-power P1 for 20 min, and then the slides were taken out to cool down to room temperature (20–23 °C). After that, the sections were washed 3 times for 3 min with PBS and blocked for 1 h at room temperature using protein block buffer (1% Bovine Serum Albumin (BSA), 0.05% Tween). After the blocking step, the sections were incubated with specific primary antibody/antibodies (Table 3) overnight at 4 °C. The next day, the sections were washed with PBS 3 times for 3 min then incubated with secondary antibodies (Jackson ImmunoResearch, Cambridge, UK) for 1 h at room temperature. After that, a PBS wash was performed; then, mounting media with 4′,6-diamidino-2-phenylindole (DAPI) for nuclei counterstaining was used and, finally, a coverslip was placed. Adropin was labeled with TRITC (red), while pancreatic peptides were labeled with FITC (green; Jackson ImmunoResearch, Cambridge, UK). Finally, the sections were examined using Nikon ECLEPS Ni fluorescent microscopy (Nikon, Shinagawa, Japan) and NIS-Element D software (Version 4.2).

The specificity of the adropin antibody was tested by using brain tissue as a positive control when immunofluorescence staining was performed. Adropin expression has been previously detected in the brain. Moreover, we incubated a peptide block with the adropin antibody in a separate experiment. The result showed no signal.

#### 4.3.2. Immunoelectron Microscopy

The tissue sections that were placed on the copper grid earlier were jet-washed with distilled water and then incubated in 10% H_2_O_2_ for 10 min, followed by a washing buffer (PBS, 1% BSA, and 0.1% Tween 20) wash, and then incubated with 0.5 M NH_4_Cl for 20 min. After that, the sections were washed again using a washing buffer and then blocked for 10 min in a blocking buffer (20% Normal Goat Serum (NGS)). Then, the sections were incubated overnight with the first primary antibody (anti-insulin antibody or anti-glucagon antibody (mouse); Table 3) at 4 °C. The next day, the sections were washed twice for 10 min using a washing buffer and then incubated with donkey anti-mouse IgG secondary antibody conjugated with 12 nm gold particles (Jackson ImmunoResearch, Cambridge, UK) for 2 h. After washing the sections with distilled water, the second primary antibody (adropin; Table 3) incubation was performed overnight at 4 °C. The next day, the sections were washed twice for 10 min using washing buffer and then incubated with goat anti-rabbit IgG secondary antibody conjugated with 6 nm gold particles (Jackson ImmunoResearch, Cambridge, UK) for 2 h. After washing and blocking the sections, the tissue sections were fixed using glutaraldehyde, washed again, and left overnight to dry. The following day, contrast staining was performed using uranyl acetate and lead citrate, and then the sections were washed and dried. The grids were examined with a Philips transmission electron microscope.

### 4.4. Adropin Levels’ Determination by Enzyme-Linked Immunosorbent Assay (ELISA)

Serum adropin levels were quantified with commercially available rat ELISA kits (Phoenix Pharmaceutical, Burlingame, CA, USA). First, standards were prepared, and then they were loaded into a 96-well immunoplate. The positive control and the samples were loaded into the plate. Primary antibody and biotinylated adropin were added and then incubated at room temperature for 2 h. The plate was washed 4 times and then streptavidin-horseradish peroxidase (HRP) was added to the wells and incubated for 1 h. The plate was washed 4 times again and then TMB was added into the wells and incubated in the dark. After 1 h, the reaction was stopped using 2 N HCL, and the absorbance was measured at 450 nm.

### 4.5. Hormone Peptide Analysis

For hormone peptide analysis, the MILLIPLEX^®^ MAP instrument, which is based on Luminex χMAP multiplex technology (Milliplex, Billerica, MA, USA), was used for the detection and quantification of a wide range of analytes, including the following hormones in serum: insulin, C-peptide, amylin, glucagon, pancreatic polypeptide (PP), peptide YY (PYY), glucagon-like peptide-1 (GLP-1), and gastric inhibitory peptide (GIP). The rat metabolic hormone magnetic beads kit was used. The standards and the serum samples were loaded into the 96-well plate. Then, the magnetic beads were added to each well and incubated overnight at 4 °C. The next day, the plate was washed, then incubated with detection antibodies for 30 min at room temperature, followed by another 30 min incubation with streptavidin–phycoerythrin. Finally, drive fluid was loaded, and the readings were recorded. The Luminex^®^ χMAP (Magpix^®^) analyzer (Milliplex, Billerica, MA, USA), which quantifies the bioassay based on fluorescent reporter signals, was used to read the 96-well plate.

### 4.6. Stimulation of Insulin and Glucagon Secretion from Cell Line and/or Pancreatic Tissue Fragments of Normal and Diabetic Rats Treated with Adropin

To investigate if adropin can stimulate insulin secretion from pancreatic β-cells, INS-1832/3, the cells were removed from liquid nitrogen, thawed, and expanded in an RPMI-1640 solution (Sigma Cat. No. R0883). The RPMI-1640 was supplemented with 2 mM L-glutamine (Cat. No. TMS-002-C), 1 mM sodium pyruvate (Cat. No. TMS-005-B), 10 mM HEPES (Cat. No. TMS-003-C), 0.05 mM β-mercaptoethanol (Cat. No. ES-007-E), and 10% FBS (Cat. No. ES-009-B). The rat insulinoma cell line (MilliporeMerk, Darmstadt, Germany) was then treated with adropin. INS-1832/3 rat insulinoma cells were seeded at a specific density, and the growth was monitored until they reached 80% confluency. The glucose-stimulated insulin secretion was performed in HEPES Balanced Salt Solution (HBSS). For the assay, the cells were washed twice in HBSS + 2.5 mM glucose and then incubated in HBSS for 1 h. After that, the cells were treated with different concentrations of adropin (10^−6^, 10^−9^, and 10^−12^ M; Phoenix Pharmaceuticals, Burlingame, CA, USA) in the presence of glucose (2.5 mM or 15 mM). After 2 h of incubation at 37 °C, the secretagogues were collected. Then, the insulin content was measured in the secretagogues using a Mercodia High-Range Rat Insulin ELISA Kit (Mercodia, Uppsala, Sweden). Furthermore, to study how adropin can alter pancreatic peptide secretion from β- to α-cells, the pancreas from normal and diabetic rats were removed and placed in ice-cold Krebs Ringer buffer. The pancreas was trimmed free of adherent fat and connective tissue and cut into small fragments. The pancreatic fragments were placed in 2 mL glass vials containing Krebs buffer and preincubated for 15 min in a water bath (37 °C) to wash away any enzymes and hormones due to cutting the tissue. After the preincubation period, Krebs buffer was drained, and the fragments were subsequently incubated for 1 h with different concentrations of adropin (10^−6^, 10^−9^, and 10^−12^ M) prepared in Krebs buffer containing 2.8 mM glucose. The control groups were incubated in Krebs buffer with no peptides. During the incubation period, each vial was bubbled with 95% O_2_ and 5% CO_2_ every 10 min. At the end of the experiment, the tissue fragments were removed, blotted, and weighted, and the supernatants were stored at –20 °C for further analysis. To measure insulin levels in the pancreatic tissue fragments supernatant, the Mercodia Ultrasensitive Rat Insulin ELISA Kit (Mercodia Developing Diagnostics, Uppsala, Sweden) was used. The Quantikine Glucagon ELISA Kit (R&D systems, Minneapolis, MN, USA) was used to measure glucagon levels in the pancreatic tissue fragments supernatant.

### 4.7. Western Blotting

Tissue homogenate of pancreas of normal and diabetic rats was prepared using RIPA buffer to obtain total protein extracts. The total protein concentration was determined using the Bio-Rad Bradford protein assay method. Then, stacking gel and running gel were prepared using 30% acrylamide gel, and then 20 μg of the tissue homogenate was denatured by heating at 95 °C for 5 min. The samples were separated by 8–10% sodium dodecyl-sulfate polyacrylamide gel electrophoresis (SDS-PAGE), and then blotted onto a 0.2 μm PVDF transfer membrane. The transfer was performed overnight at 30 v. After that, the protein bands on the PVDF membrane were blocked using 3% non-fat milk, followed by overnight incubation with specific primary antibody raised in rabbit for GRP19 (Origene Biotechnologies, Rockville, MD, USA) at 4 °C. The next day, the membrane was washed with Tris-buffered saline (TBS) containing Tween 20 and then incubated with anti-rabbit IgG HRP-labeled secondary antibody (BIO-RAD, Hercules, CA, USA) at RT (20–23 °C) for 1 h. Finally, the protein bands were detected with an enhanced chemiluminescence (ECL) Western blotting substrate kit (ThermoFisher, Norristown, PA, USA). GAPDH was used for normalization.

### 4.8. Quantification of Images

For cell counting of the histological images and band quantification for the Western blotting technique, ImageJ (1.53) software was used. To quantify the immuno-staining of the pancreas, the number of labeled cells with specific antibodies was counted using the Cell Counter plugin in the software. First, the total number of cells in the pancreatic islet was counted (counting DAPI-labeled nuclei); then, cells labeled with specific immunofluorescence secondary antibody were counted. After that, the percentage of specific immunofluorescence secondary-antibody-labeled cells with respect to the total number of cells was calculated.

In Western blotting technique, band intensities of the target protein as well as the housekeeping gene were quantified, then normalization was performed.

### 4.9. Statistical Analysis

The statistical analysis was performed using GraphPad Prism (9.4.1) software to compare the means of the different groups. The data were analyzed via the independent Student’s *t*-test and ANOVA test. Tukey’s post hoc test for multiple comparisons was performed. Data were expressed as means ± SEM. *p*-values < 0.05 were considered as significant.

## 5. Conclusions

The results demonstrated that adropin treatment inhibited glucagon secretion from pancreatic α-cells in diabetic rats and pancreatic tissue fragments. However, adropin administration did not affect serum insulin levels in diabetic rats or insulin release from INS-1 832/3 rat insulinoma cells and pancreatic tissue fragments. Additionally, adropin did not have the ability to restore pancreatic β-cells. Our findings suggested that the adropin peptide may play a role in modulating glucagon secretion in an animal model of diabetes mellitus, offering a potential therapeutic target for this chronic disease.

## 6. Limitations of the Study and Future Contexts

Although we showed some promising results, some limitations in this study should be addressed. First, the chemically induced diabetic rat was the animal model of diabetes mellitus used in this investigation. A single large dose of 60 mg/kg of STZ was administered. STZ is a chemical substance that targets pancreatic β-cells and destroys them, causing insulin-dependent-like diabetes. In spite of that, this model of T1DM does not bear strong autoimmune features, as seen in the human version of the disease. Therefore, animal models of spontaneous (genetically modified) T1DM, such as NOD mice, where autoimmunity is involved in β-cell destruction, could be used in future studies. Additionally, in our study, adropin was administered for 10 days, based on the literature. However, there was no change in fasting blood glucose levels between diabetic untreated in comparison to diabetic treated rats, unlike what was reported in several other studies, which demonstrated that adropin can reduce fasting blood glucose in diabetic animals. Thus, to attain a more chronic effect, increasing the duration of adropin administration and running HbA1c tests should be considered. Moreover, we reported a decrease in the α-cell number and glucagon release, as well as a significant increase in serum GLP-1 levels, so to further investigate the effect of adropin on pancreatic α-cells and how it could modulate glucagon secretion and/or action, an in vitro study can be conducted using the αTC1-9 cell line. This cell line is well differentiated and produces only glucagon, and it is useful for investigating glucagon biosynthesis and α-cells’ sensitivity to cytokines. Furthermore, to expand our understanding of the adropin mechanism involved in modulating glucagon and GLP-1 secretion in T1DM, proglucagon cleavage involving prohormone convertase 1 and 2 expression could be studied in a T1DM animal model treated with adropin. Further mechanistic investigations using a variety of techniques, including genomics, transcriptomics, proteomics, and metabolomics, to decipher the nature of the metabolites and signaling pathways [69,70], could help to gain a better understanding of the mechanism by which adropin exerts its actions.

## Figures and Tables

**Table 1:** Summary of the main results.

Adropin Expression in Pancreatic Endocrine Cells in Normal and Diabetic Rats		Effect of Adropin on Serum Levels of Insulin, Glucagon, PP, PYY, GIP, GLP-1, and Amylin in Normal and Diabetic Rats	
β-cells	Expressed in normal and diabetic rats	Insulin and PP	No change
α-cells	Expressed in normal and diabetic rats	Glucagon	Decreased (*p* > 0.05)
PP-cells	Expressed in normal and diabetic rats	PYY and GIP	No change
D-cells	Expressed in normal and diabetic rats	GLP-1 and amylin	Increased (*p* < 0.05)

PP = pancreatic polypeptide; PYY = peptide YY; GLP-1 = glucagon-like peptide 1; GIP = gastric inhibitory peptide.

**Table 2:** Animal groups and adropin treatment.

Group	Treatment
Normal untreated (NUT; *n* = 6)	Vehicle (PBS)
Normal treated (NT; *n* = 6)	Adropin (dose: 2.1 μg/kg/day)
Diabetes mellitus untreated (DMUT; *n* = 6)	Vehicle (PBS)
Diabetes mellitus treated (DMT; *n* = 6)	Adropin (dose: 2.1 μg/kg/day)

**Table 3:** Primary antibodies for immune-histological analysis.

Primary Antibodies	Dilution	Company
Adropin (rabbit)	1:100	Thermo Fisher, Waltham, MA, USA
Insulin (guinea pig)	1:2000	Dako, Glostrup, Denmark
Glucagon (mouse)	1:2000	Abcam, Waltham, MA, USA
Somatostatin (mouse)	1:500	Thermo Fisher, Waltham, MA, USA
Pancreatic polypeptide (goat)	1:100	Abcam, Waltham, MA, USA

## Data Availability

All available data have been provided in the manuscript.

## Supplementary Materials

The following supporting information can be downloaded at: https://www.mdpi.com/article/10.3390/ijms25189824/s1.
