# Supplementary material for: Adropin Is Expressed in Pancreatic Islet Cells and Reduces Glucagon Release in Diabetes Mellitus
Source: Int J Mol Sci. 2024 Sep 11;25(18):9824. doi: 10.3390/ijms25189824 (PMC11432804; doi:10.3390/ijms25189824)
Supplement: Supplementary file 1 [file ijms-25-09824-s001.zip › ijms-3144527-supplementary.pdf]

Supplement figures

Supplement Fig S1

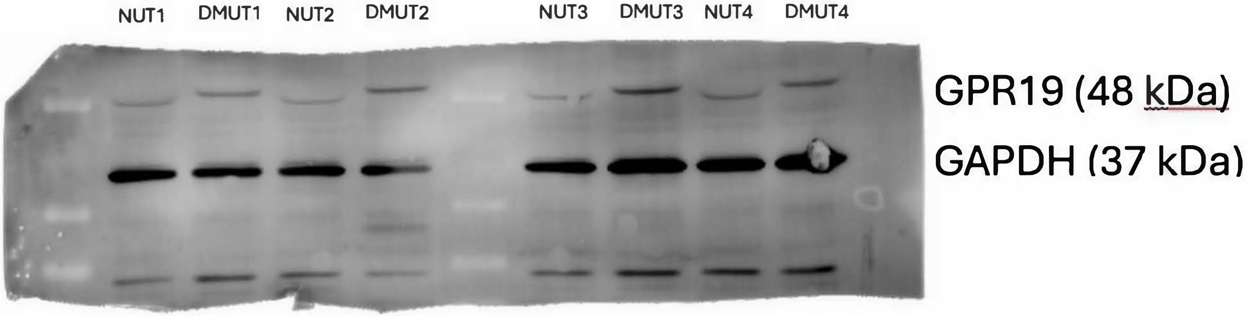

Figure S1. Uncropped raw Western blots of GPR19 and GAPDH in the pancreas of normal and diabetic rats.
